# Emerging Strategies in Drug Development and Clinical Care in the Era of Personalized and Precision Medicine

**DOI:** 10.3390/pharmaceutics16081107

**Published:** 2024-08-22

**Authors:** Cristina Manuela Drăgoi, Alina Crenguța Nicolae, Ion-Bogdan Dumitrescu

**Affiliations:** Faculty of Pharmacy, “Carol Davila” University of Medicine and Pharmacy, 020956 Bucharest, Romania; cristina.dragoi@umfcd.ro (C.M.D.); ion.dumitrescu@umfcd.ro (I.-B.D.)

## 1. Introduction

In the ever-changing landscape of modern medicine, we face an important moment where the interplay of disease, drugs, and patients defines a new paradigm. The trajectory is shifting from conventional drug development towards personalized therapeutic strategies addressed to the unique physiology of each individual. In recent decades, remarkable efforts in preventive measures, diagnostic techniques, and treatment approaches have led to substantial enhancements in patient care. However, the achievement of personalized medicine strongly depends on a comprehensive understanding of pathogenesis, therapeutic agents, biochemical mechanisms, drug interactions, and patient-specific factors [1,2,3].

In this setting, the aim of this Special Issue on “Emerging strategies in drug development and clinical care in the era of personalized and precision medicine” was to assemble a compendium of the most recent pertinent research papers elucidating the current state-of-the-art knowledge and projecting future directions in drug development and clinical practice. Central to this effort was the focus on biochemical mechanisms of action, innovative drug formulations, and rigorous preclinical and clinical evaluations encompassing efficacy, pharmacokinetics, and toxicity within the framework of precision and personalized medicine.

This Special Issue addressed a significant topic by synthesizing cutting-edge research across various facets of drug development and clinical care. By consolidating insights into therapeutic strategies, drug design, and pharmacological testing, it offers a detailed pathway for crossing the bridges of medicine to the new horizons of personalized medicine. Moreover, it underscores the imperative of interdisciplinary collaboration and the integration of diverse data sources to unlock new avenues for therapeutic innovation, as in recent years, the integration of smart wearables and artificial intelligence (AI) technology into healthcare has revolutionized personalized medicine [4,5,6]. These advancements have the potential to enhance patient outcomes, improve disease management, and offer more tailored therapeutic interventions.

The significance of these technologies in personalized medicine and their transformative impact on the healthcare landscape comes not only from helping individuals maintain a healthier lifestyle, but also from continuously providing physiological and metabolic data essential for managing chronic diseases. They enable continuous and real-time monitoring of an individual’s health status, gathering extensive data on various physiological and metabolic parameters, such as heart rate, blood pressure, glucose levels, physical activity, and sleep patterns. The integration of these data with AI algorithms allows for the development of highly personalized health interventions tailored to the specific needs of each individual [7,8].

## 2. A Glimpse over the Published Studies

The field of personalized and precision medicine is experiencing a profound transformation, marked by innovative approaches for drug development and clinical care. In this Special Issue of *Pharmaceutics*, thirteen groundbreaking papers delve into diverse aspects of this evolving landscape, shedding light on novel therapeutic strategies, molecular mechanisms, and diagnostic methodologies. Through rigorous investigation and interdisciplinary collaboration, these studies contribute significantly to our understanding of personalized medicine and pave the way for future advancements in patient care.

One notable study by Dräger et al. investigates the impact of model-informed precision dosing on procalcitonin concentrations in critically ill patients. By using advanced dosing strategies, the authors demonstrate the potential to optimize antibiotic treatment and improve patient outcomes. Similarly, Kwak et al. employ molecular networking techniques to elucidate the metabolic pathways of anamorelin, offering valuable insights into the pharmacokinetics and pharmacodynamics of this growth hormone secretagogue receptor agonist.

In another study, Joung et al. approached the therapeutic potential of selegiline in modulating lipid metabolism in obese mice, highlighting the importance of targeting specific molecular pathways in the management of metabolic disorders. Furthermore, Kanellopoulos et al. propose structural interventions to enhance the stability of radioligands targeted at the neurotensin subtype 1 receptor, laying the groundwork for more effective cancer theranostics.

This Special Issue also features population pharmacokinetic analyses of perampanel in patients with refractory epilepsy by Silva et al., as well as investigations into the effects of angiotensin II receptor blockers on cerebrospinal fluid biomarkers in Alzheimer’s disease by García-Lluch et al. These studies underscore the growing emphasis on individualized treatment approaches tailored to the unique characteristics of each patient.

Moreover, Becker et al. explore the role of kinin receptors in cisplatin-induced peripheral neuropathy, while Axente et al. provide insights into the clinical and electrophysiological changes in pediatric spinal muscular atrophy following nusinersen treatment. These findings contribute to a better understanding of neurological disorders and the targeted therapeutic interventions employed by clinicians.

Additionally, this Special Issue features comprehensive reviews by Marques et al., Sánchez Suárez et al., Rakicevic, and Walker et al., which offer comprehensive overviews of innovative in silico approaches for drug development, personalized treatment strategies for chronic lymphocytic leukaemia, nucleic acid-based therapies for cardiovascular diseases, and the diagnostic applications of exosomes in muscle-invasive bladder cancer, respectively.

In the last article published in this Special Issue, Anne Harnett et al. present a comprehensive study on the prevalence and management of swallowing difficulties among acute hospital inpatients. This survey reveals that a great number of patients face challenges swallowing solid oral dose forms, with significant implications for medication administration safety and efficacy. The authors emphasize the risk of inappropriate modifications, which can lead to patient harm. They advocate for a proactive approach, such as implementing screening tools, to identify patients with swallowing difficulties and ensure safer medication administration practices.

## 3. Novel Biomarkers in Precision Medicine

Personalized medicine represents a paradigm shift in healthcare, focusing on tailoring medical treatment to the individual characteristics of each patient. Central to this approach is the identification and application of novel biomarkers—biological molecules that serve as indicators of a biological state or condition. These biomarkers can be found in blood, tissues, or other bodily fluids, and their discovery and validation are crucial for advancing personalized medicine [9,10,11,12,13,14].

One of the key advantages of novel biomarkers in personalized medicine is their potential to enhance the specificity and sensitivity of disease diagnosis. Traditional diagnostic methods often rely on generalized criteria that may not account for individual variations in disease presentation [15,16,17]. Novel biomarkers enable the detection of diseases at earlier stages and with greater accuracy by reflecting the unique molecular signatures associated with different disease states. For example, the identification of specific genetic mutations, such as BRCA1 and BRCA2 in breast cancer, has revolutionized the screening and risk assessment for patients, allowing for more targeted and effective interventions [18].

Moreover, novel biomarkers play a pivotal role in the customization of therapeutic strategies. In cancer treatment, for instance, the expression levels of certain proteins or the presence of specific genetic alterations can guide the selection of targeted therapies, thereby improving treatment efficacy and minimizing adverse effects. The development of companion diagnostic tests that identify the suitability of a particular therapy for a patient based on their biomarker profile exemplifies this approach [19,20,21]. Personalized treatment plans based on biomarker information can lead to better outcomes, as seen with the use of HER2 inhibitors in HER2-positive breast cancer patients [22].

In addition to their diagnostic and therapeutic applications, novel biomarkers are essential for monitoring disease progression and treatment response. Biomarkers can provide real-time insights into how a disease evolves and how a patient responds to treatment, enabling dynamic adjustments to therapeutic regimens. For chronic patients with diabetes, cardiovascular, neurologic and psychiatric diseases, osteoporosis, inflammatory and autoimmune conditions, there are several biomarkers that offer valuable information on disease control and risk stratification, facilitating more proactive and individualized management strategies [14,23,24,25,26,27,28,29,30].

The future of personalized medicine hinges on the continuous discovery and validation of novel biomarkers. Advances in high-throughput technologies, such as next-generation sequencing and mass spectrometry, have accelerated the identification of potential biomarkers. Integrative approaches combining genomics, proteomics, metabolomics, and other omics data are poised to uncover a comprehensive array of biomarkers that reflect the complexity of human diseases. As our understanding of the molecular underpinnings of diseases deepens, the translation of these biomarkers into clinical practice will further refine and revolutionize personalized medicine, ultimately leading to more precise, effective, and patient-centered healthcare [2,19,31].

## 4. A Preview of AI-Integrated Technologies for Shaping the Future of Personalized Medicine

The nine research studies and four reviews featured in this Special Issue comprehensively outline the current practices, highlight gaps in solution-finding approaches, and present future perspectives in the field of personalized medicine. A significant advancement in this area is the integration of technology, particularly the application of artificial intelligence (AI) in patient care. Smart devices are capable of continuously collecting data on vital signs and other health metrics, providing a comprehensive and real-time overview of an individual’s health status. This continuous monitoring facilitates the early detection of potential health issues, thereby enabling timely interventions and enhanced disease management [32,33].

For individuals with chronic conditions such as metabolic, cardiovascular and respiratory diseases, wearable devices offer a practical solution for ongoing health monitoring. AI algorithms play a central role by analyzing the collected data to identify trends and anomalies, allowing healthcare providers to adjust treatment plans accordingly. This data-driven approach not only improves patient outcomes but also optimizes the overall management of chronic diseases.

AI-driven analysis of data from smart devices provides personalized insights into an individual’s health behaviors and their impacts. This can help people make informed decisions about their lifestyle, such as adjusting their diet, exercise routine, or medication adherence, in order to optimize their health. Another important role is in acknowledging the impact of every small intervention regarding nutrition, sleep patterns, and chronobiologic approaches intended to optimize activity versus rest outlines, on the general state of health for an individual [34,35,36,37,38,39,40].

By employing machine learning and predictive analytics, AI can identify patterns and predict future health risks. This proactive approach allows for the implementation of preventive measures, potentially reducing the incidence and severity of chronic diseases.

Smart devices also encourage patients to take an active role in managing their health. The feedback provided by these devices can motivate users to adhere to healthier behaviors and treatment plans, fostering a sense of empowerment and responsibility for their own well-being. The data collected by wearable devices can be integrated with electronic health records, providing healthcare professionals with a more comprehensive view of a patient’s health. This holistic approach facilitates more accurate diagnoses, personalized treatment plans, and improved coordination of care.

The vast amounts of data generated by smart devices contribute to medical research by offering insights into population health trends and the effectiveness of various interventions. These data can inform the development of new treatments and healthcare policies aimed at improving public health [41].

## 5. Conclusions

In conclusion, the journey towards personalized and precision medicine represents a paradigm shift in healthcare, with the potential to revolutionize patient care and outcomes. This Special Issue serves as a reassurance statement, revealing the path for the future where therapeutic interventions are not only efficacious but also intricately tailored to the unique needs of each patient. As the next step forward, the integration of smart devices and AI into personalized medicine represents a significant advancement in healthcare. These technologies enable continuous health monitoring, personalized nutritional, lifestyle, chronobiological and medical interventions, and predictive analytics, which collectively enhance disease management, improve patient outcomes, and promote proactive health management.
